# The ethics of caring for hospital-dependent patients

**DOI:** 10.1186/s12910-017-0238-1

**Published:** 2017-12-11

**Authors:** Calvin Sung, Jennifer L. Herbst

**Affiliations:** 1 Frank H. Netter MD School of Medicine at Quinnipiac University, 275 Mount Carmel Ave, Hamden, CT 06518-1908 USA; 2 Quinnipiac University School of Law, 275 Mount Carmel Ave., Hamden, CT 06518-1908 USA

**Keywords:** Hospital-dependent patient, Acute care hospital, Length of stay, Palliative care, Hospitalist, Intensivist, Clinical ethics, Professional obligations, Role morality, Dual agency

## Abstract

**Background:**

Hospital-dependent patients are individuals who are repeatedly readmitted to the hospital because their acute medical needs cannot be met elsewhere. Unlike the chronically critically ill, these patients do not have a continuous need for life-sustaining equipment and can experience periods of relative stability where they have a good quality of life. However, some end up spending months or even years in the hospital receiving resource-intensive care because they are unable to be safely discharged, despite an initial optimistic prognosis. It is hard to reliably identify these patients on admission and more research is needed to better understand the unique medical needs of this population. But the inability to safely discharge these patients to their home or to a skilled nursing facility without rapid readmissions also creates ethical implications for the physicians who care for them. The aim of this paper is to clarify some of the ethical considerations involved in caring for hospital-dependent patients.

**Main body:**

Among physicians, the care of hospital-dependent patients is likely to disproportionately affect hospitalists and intensivists, whose care is often evaluated in terms of reducing patient length of stay and readmissions. Because hospital-dependent patients’ medical needs thwart the traditional goal of safe discharge, both clinical ethics and physicians’ professional obligations are implicated by their care. The inability to reliably identify these patients early can complicate discussions about treatment goals and informed consent. Similarly, the tremendous dedication of limited resources to these patients without safe discharge back to the community may raise concerns about the just allocation of healthcare resources.

**Conclusion:**

Our current acute care hospitals are not designed to provide long-term care for hospital-dependent patients. Unfortunately, safe discharge options remain elusive for these patients. Further research and support of this population is needed to more reliably identify hospital-dependent patients on admission, better inform the discussions of short- and long-term treatment goals, and more wisely allocate resources both within our acute care hospitals and larger healthcare system.

## Background

Acute care hospitals are designed to quickly treat illness and disease so that patients can be discharged back into their communities [1]. Patients are meant to stay only until they no longer need the level of care uniquely provided in the hospital setting [2]. Some patients experience repeated hospitalizations with short duration out of the hospital and risk spending months or years in intensive care and medical units despite high quality inpatient care, comprehensive discharge planning, and adherence to outpatient follow-up [3, 4]. These “hospital-dependent patients” (HDPs) are individuals whose medical conditions require the high staffing ratios, monitoring and diagnostic capabilities, and quick response by on-site care teams only available in a hospital [5].

Compared to the more easily identified chronically critically ill patients, HDPs may not appear as sick and do not require continuous life-sustaining equipment. HDPs may even have periods of relative stability where they are able to have an acceptable quality of life for themselves in the hospital. Even so, their complex and extensive healthcare needs, in combination with an unexpected lack of resilience, leave them dependent on the hospital setting where services are the most expensive and resources are limited. Most HDPs are government-insured, and thus often reimbursed below cost, especially for lengthy inpatient stays. In addition, hospitals face potential financial penalties for higher-than-expected readmissions, further reducing reimbursement [6]. Taken together, this means acute care hospitals struggle to provide cost-effective care for these patients.

Very little is known about HDPs even though they are thought to have existed for many years [5]. As yet, there is no consensus on how to prospectively identify a HDP. Current prognostic indicators available to most physicians do a poor job of predicting the health outcomes of HDPs and large systematic studies are needed to develop a better understanding of their needs. This paper attempts to clarify some of the ethical considerations that may be relevant in caring for HDPs.

## Main text

Many advances in medicine and public health have increased our ability to manage chronic conditions and sustain life. While our improved ability to resuscitate, stabilize, and manage patients is no doubt a welcome achievement, medicine unfortunately still struggles to return HDPs to their lives in the community [7]. A HDP typically arrives at a hospital due to a crisis such as a myocardial infarction, pulmonary edema, sudden delirium, or fall, and may also suffer from multiple chronic conditions [5]. Because their multiple chronic conditions make them more fragile, HDPs are more likely to be admitted as inpatients. In addition to the inherent risks of medical care, hospital admission itself may lead to further loss of ability to perform basic self-care activities that are fundamental to maintaining independence and quality of life [8]. Some patients will continue to require the ongoing services of acute care hospitals and may not be able to receive adequate care elsewhere. For example, they may require the extensive lab capabilities, imaging modalities, and highly-skilled nursing services that are found in acute-care hospitals. Long-term care hospitals, on the other hand, are frequently specialized to care for patients with persistent organ failure and a continuous need for life-sustaining equipment, such as prolonged mechanical ventilation, which HDPs do not have [5, 9]. HDPs tend to be best served in acute care hospitals as illustrated by the following hypothetical cases.

John Smith is a 74-year-old male with a history of congestive heart failure, atrial fibrillation, myocardial infarction, and status post-coronary artery bypass graft who presents to the hospital with a heart failure exacerbation. He is treated with diuretics for symptomatic relief but subsequently develops orthostatic hypotension as a result of excessive fluid loss. On his way to the bathroom John falls and fractures his hip. He is sent for a hip replacement surgery and spends an additional 5 days in the hospital. After recovering from surgery he is discharged to a rehabilitation facility only to be found back at the hospital two weeks later for another unexpected fall. X-ray confirms a pelvic fracture and John ends up spending 2 more nights on the medical floor while his care team attempts to manage his pain and correct his orthostatic hypotension. He is discharged back to the same rehabilitation facility but returns to the hospital a week later after another fall while attempting to stand up from the toilet. He is stabilized in the hospital but is now fearful of falling again and his care team is reluctant to discharge him.

Jane Doe is an 82-year-old female with a history of recurrent urinary tract infections, chronic back pain, diabetes, and hypertension who presents to the hospital in septic shock. She is immediately given vasopressors to raise her blood pressure and started on empiric antibiotic therapy. Though her circulation improves, Jane experiences a reaction to the antibiotics which causes acute kidney injury and is admitted to the ICU. She is started on emergency dialysis which also limits the number of drugs that can be safely administered for her other conditions. In particular, her physicians struggle to manage her increasing back pain with opioids while maintaining the blood pressure necessary for dialysis. After several weeks in the ICU Jane eventually dies from irreparable kidney damage.

Both John and Jane are hypothetical elderly individuals with multiple chronic conditions. Neither were admitted with a diagnosis of terminal illness or required continuous life-sustaining equipment. John experiences a prolonged hospital stay after his fall and requires further imaging and surgery. Once he is discharged to the rehabilitation facility, he is likely more fragile than when he first entered the hospital and, as a result, more prone to future injuries even if he adheres to all recommended therapy and prescriptions. Jane, on the other hand, has a history of multiple hospitalizations due to her urinary tract infections and is treated with interventions that are typically only found in hospitals such as vasopressors and intravenous antibiotics. Her kidney injury likely prompts a nephrology consult and she requires close monitoring in the ICU for careful titration of her medications. Her physiologic reserve becomes compromised as a result of many complications of her hospital stay until she eventually dies in the hospital.

Despite the many sudden and unexpected decompensations characteristic of HDPs, many may be transiently stabilized enough to maintain a quality of life in the hospital that is acceptable to them [5]. When admitted as an inpatient, HDPs may be relieved of the burden of many decisions that can become tedious or increasingly difficult for those navigating ill health—what to eat, what to wear, what to clean, what to fix, who to call, who (if anyone) to see, how to navigate a spousal/parental/sibling relationship now disrupted by intimate health concerns. For HDPs with limited or dwindling social networks, admission to a hospital may provide social interaction that was otherwise difficult to sustain or create [10]. For some HDPs, the reduced decisional burden and increased social contact may outweigh the loss of control they experience as an inpatient. As a result, many of these patients can end up spending significant periods of time in the hospital, especially when they feel more secure and connected [5] and hospitals are likely to risk financial penalties created by the Affordable Care Act for their readmission after yet another rapid post-discharge decompensation. These penalties may be triggered when Medicare patients with principal discharge diagnoses of acute myocardial infarction, heart failure, pneumonia, chronic obstructive pulmonary disease, elective total hip and/or total knee arthroplasty, or coronary artery bypass graft surgery are readmitted to the hospital for any reason within 30 days of their discharge [11].

While caring for HDPs may present a challenge for any clinician who may encounter them, physicians whose practice is spent primarily or entirely caring for patients in medical units and ICUs (for example, hospitalists and intensivists) may be disproportionately affected because of the increased attention on reducing patient length of stay and readmissions [12–17].

All physicians, hospitalists and intensivists included, are expected to abide by the principles of clinical ethics and the obligations shared by their professional bodies. Traditionally, bioethics literature has focused on the clinical ethics of delivering appropriate care to patients [18]. But to the extent that professional obligations frame community expectations and professional identity (which in turn affects patient care) [19–21], physicians’ obligations to their profession are also important to consider [22, 23]. HDPs raise questions for physicians in clinical ethics and challenge some of their professional obligations.

### How should doctors respond and provide clinical care to HDPs?

A large body of bioethics literature has framed the core values of clinical medicine in terms of the principles of beneficence, nonmaleficence, respect for autonomy, and social justice [24]. Physicians can look to these principles to navigate ethical issues that may arise in caring for their patients.

The principles of beneficence and nonmaleficence require any harm, pain, discomfort, or suffering caused by medical care to be outweighed by the benefit to the patient. Historically, many physicians and patients assumed that more care was inherently better care. More recently, though, both physicians and patients have questioned whether some acute care treatments are justified due to significant risks and unlikely or unclear benefit, especially when compounded by the health risks associated with hospitalization. Prolonged hospital stays are associated with increased risks of morbidity and mortality [25]. The risk of nosocomial infection, for example, may threaten patient safety [26] enough to prompt swift transfer once a patient’s immediate health needs have been met. However, once an HDP has been identified, the likelihood of rapid decompensation (despite current stabilization) may prevent them from being transferred to a lower level of care in the interest of reducing readmissions.

Many (but certainly not all) physicians question the quality of living in an acute care hospital and plan their own end-of-life care to minimize the time they spend as an inpatient [27–29]. HDPs, however, are so prone to crisis that they may feel more secure surrounded by the unique resources of a hospital and have an acceptable quality of life while they are transiently stabilized [5]. This may appear to be a conflict between nonmaleficence on the one hand, i.e., swift transfer of a stabilized patient out of the hospital meant to reduce the risks associated with a prolonged stay, and patient autonomy on the other hand, where a patient requests to stay in the hospital. For most patients, the risk of prolonged hospitalization means that the patient’s request to remain an inpatient is potentially inappropriate [30]. However, it isn’t clear whether the risk-benefit analysis of hospitalization is representative of HDPs. It may be better for HDPs to remain in the hospital, but without more data nonmaleficence remains a concern.

Because the acute hospital setting necessarily limits patient choice, especially in the course of treating a series of emergent medical crises, caring for HDPs may provoke a sense that medical care is undermining patient autonomy. While many advances in medicine have expanded our capabilities to treat and manage hospitalized patients, it has also created a population of patients who become dependent on repeated acute care (and thus, highly time-sensitive) treatments to stay alive [5]. Ideally, the informed consent process anticipates that physicians and patients (or their surrogate decision-makers) jointly consider each patient’s diagnosis and proposed treatment, the proposed treatment’s risks and benefits, alternative procedures and their risks and benefits, and the risks and benefits of taking no action [31]. In the context of caring for HDPs, however, rapid decompensation and the need for immediate care may result in repeated use of “presumed” consent, where it is presumed that reasonable people would consent to treatment in life-threatening situations [32, 33].

The acuity of an emergency situation certainly does not inherently preclude the informed consent process, but it can lead clinicians to make quick judgments about their patients that may not be true. As an example, for some acutely ill patients, physicians may conflate consciousness with mental capacity and mistakenly presume that a conscious patient is able to properly consent to treatment. In addition to being conscious, having mental capacity means to understand, retain, and use information with regards to a particular situation and communicate a decision. Without explicitly assessing for mental capacity, clinicians sometimes fail to recognize that conscious patients are in fact cognitively impaired [34]. But even in situations where HDPs or their surrogates have capacity to make decisions, physicians may still struggle to provide meaningful information about the potential risks and benefits of proposed care because HDPs remain difficult to identify proactively.

The lack of knowledge about HDPs challenges the physician’s commitment to professional competence. HDPs may have multiple chronic illnesses on admission, but are generally expected, based on similarly appearing patients, to be restored to their usual health after addressing their acute stress or injury [5]. It may not be apparent until the next admission that the standard of care for most patients will not have the same therapeutic results for HDPs because of their unanticipated fragility. In an emergency setting where it is not always possible to communicate effectively with patients, physicians rely on a standard of care mutually agreed upon by colleagues to guide treatment decisions. Since we are not yet successful at distinguishing HDPs on admission it is difficult to tailor treatment to their specific needs. The standard of care that is normally successful in discharging patients may not be appropriate for HDPs. Therefore, when HDPs return to the hospital, the physicians and interprofessional care teams assigned to their care may be implicated for failing to discharge them safely.

Part of the challenge remains that HDPs are difficult to identify (at least initially) and frequently present similarly to patients who can be managed successfully with non-acute outpatient support. To the extent that HDPs find relief in the acute care setting due to the logistical, financial, or relational burden that ill health can create (or exacerbate), palliative care may be a useful tool to not only provide comfort and mobilize additional supportive services but especially to also clarify values so that HDPs can be effective agents of their own care [35]. Unfortunately, HDPs may not trigger a palliative assessment as quickly as patients more readily identified upon admission as nearing the end of life, especially where palliative care is mistakenly equated exclusively with hospice. While terminal illness status is an important factor in initiating a conversation about end-of-life goals, prognosis alone should not be the basis of palliative referrals or discussing treatment goals in the face of serious illness [36]. Rather, a holistic view of the patient’s needs should be considered in making care decisions. Given the reluctance of many physicians to discuss end-of-life treatment goals with patients who do not yet have a terminal diagnosis and the difficulty of identifying HDPs on admission [37, 38], many HDPs and their surrogates may not be making informed decisions consistent with their wishes that could improve their quality of life and help ensure that treatment is more aligned with their values. While some suggest that these conversations should be happening with every patient admitted with serious and life-threatening illness [36], HDPs may be left out of these conversations because they present healthier than they actually are.

The principle of social justice is another important consideration for physicians, but inherently difficult to consider from a clinical perspective. Unlike the principles of autonomy, beneficence, and nonmaleficence, social justice requires physicians to think beyond their patients as individuals, but rather as members of the larger community [39]. High cost interventions (especially those that still result in significant ongoing morbidity) implicate the professional and ethical tension of “dual agency” -- physicians are caught between (1) the best interests of individual patients, and (2) just distribution of healthcare resources [40]. Whether faced with a specific question of the patient “most deserving” of the last ICU bed or a larger system question where high-cost interventions for HDPs make insurance and access to healthcare impossible for others, many physicians struggle to live up to these two core tenets of professionalism. [23, 40] Attempts to implement social justice in practice, especially when physicians are being “urged to exercise judicious financial stewardship,” and still maintain consistent, unbiased, and personalized patient-centered care, can be difficult [41].

Physicians may reconcile these competing obligations by “defining different roles and spheres where the different expectations of professionalism are more or less operative,” an approach called “role morality” [40]. Individual physicians may occupy multiple roles including provider, educator, administrator, researcher, policymaker, and public health official. Each of these roles carries with it a different primary interest or obligation, e.g., the provider is focused primarily on the individual patient, the educator on the student, the administrator on the organization, the researcher on the larger body of knowledge, and the policymaker and public health official on the larger community. The specific decisions and tools in each role may be guided by the relevant professional principles and commitments while a physician is wearing that proverbial hat [40]. In other words, while treating patients, a physician may be guided by the principle of patient welfare; later, that same physician, as an administrator, may work on a system level to create and implement policies that improve the just allocation of healthcare resources.

Social justice is often understood to require equitably distributing the burdens and benefits of care that are borne by the community. Physicians observe the seemingly disproportionate amount of time, resources, and care that HDPs need, and the many other patients who go without receiving adequate care. Increasingly, physicians recognize that healthcare resources are limited and that care delivered to one patient may be preventing care from being delivered to another. Very costly care with little likelihood of successful discharge may indicate that resources are not being invested in the most effective manner to meet the health needs of the overall community. The care provided to HDPs may be considered unjust and unsustainable considering the needs of so many others who are denied the care needed to keep them productively engaged in the community. Even so, it is not clear what can be accomplished in changing the practices of individual physicians that would address the injustices seen at a community level.

“Physicians have never performed all possible tests and treatments for patients and, in this sense, have always limited care based on professional judgment. Yet questions of whether they ought not to perform indicated tests or treatments they deem too costly have vexed the profession for decades” [42]. Physicians treating HDPs may question whether they should be performing expensive tests and treatments unlikely to lead to discharge. While both clinical ethics and professional obligations ask physicians to consider the impact of their recommendations on the just allocation of resources, there remains significant concern over physicians using “bedside rationing” in the name of social justice. [41, 43] This type of ad hoc allocation has historically led to discriminatory and biased methods, resulting in distrust of the healthcare system [41]. But beginning to identify HDPs earlier and better understand their needs may start to inform system change or a modification in the standard of care to reduce the ethical tension that physicians experience when they see care as being wasteful. Individual hospitals, healthcare systems, and professional medical societies, specifically those in internal medicine and hospital medicine, could encourage more HDP-related research by providing funding, access to hospital- or system-level data, and administrative support.

## Conclusions

Hospital-dependency is a phenomenon created by the many medical advances that have helped meet the acute needs of patients. Medicine’s scientific approach to patient care has helped make incredible strides in developing evidence-based treatments for disease. But there are many complex patients whose needs continue to fall beyond the findings of our current research. The increasing pressure to lower costs of care emphasizes the need to provide more cost-effective and meaningful care for HDPs. The way we currently treat HDPs is becoming increasingly unsustainable and raises questions of our dedication to social justice and professional ethics. While role morality may allow physicians treating HDPs to ignore the costs borne by the larger healthcare system in the short term, it unfortunately is unlikely to improve the just allocation of healthcare resources unless and until the system itself is significantly reformed. More research is needed to better understand this patient population. Until then, however, physicians may be tempted to limit the costly options presented as part of the informed consent process in an attempt to promote a more just allocation of healthcare resources. This type of allocation or rationing should not happen at the individual bedside as this could lead to biases and mistrust that have contributed to health disparities. Rather, facility or system level reform could help balance the health needs of HDPs with those of the larger community.

## Abbreviation

HDP: Hospital-dependent patient
